# Chromosomal instability as a predictive biomarker for recurrence risk following transurethral resection of NMIBC

**DOI:** 10.3389/fonc.2026.1752078

**Published:** 2026-03-11

**Authors:** Qi Ding, Hailiang Zhu, Bo Fan, Lisheng Wang, Xiaohua Jin, Cheng Cao, Ying Shi, Zhijiang Fan, Wenjian Tu, Feng Li

**Affiliations:** 1 Department of Urology, The First People’s Hospital of Changshu, The Changshu Hospital Affiliated to Soochow University, Changshu, China; 2 Department of Gastroenterology, The First People’s Hospital of Changshu, The Changshu Hospital Affiliated to Soochow University, Changshu, China

**Keywords:** biomarker, bladder cancer, chromosomal instability, low-coverage whole genome sequencing, recurrence

## Abstract

**Objective:**

To investigate chromosomal instability (CIN) in tumor tissues from transurethral resection of bladder tumor (TURBT) and evaluate its feasibility as a molecular biomarker for prognostic stratification in non-muscle-invasive bladder cancer (NMIBC).

**Methods:**

In this retrospective single-center cohort study, DNA was extracted from formalin-fixed paraffin-embedded (FFPE) samples of 50 BC patients using the Qiagen nucleic acid extraction kit. The recurrence defined in this study refers to cystoscopically visible recurrence confirmed by biopsy or surgical pathology. All patients underwent follow-up until January 2025 in strict accordance with the protocol established by the EAU guidelines for NMIBC. Low-coverage whole-genome sequencing (LC-WGS) was performed to analyze CIN in bladder tumors. Time-dependent Receiver Operating Characteristic (ROC) analysis was performed to evaluate the predictive efficacy of CIN values for post-TURBT outcomes. Survival analysis was conducted using the Kaplan-Meier method (log-rank test), and associations between variables and recurrence-free survival (RFS) were assessed via Cox proportional hazards models.

**Results:**

Recurrent bladder cancer patients exhibited significant copy number alterations (CNAs) differences in 67 gene segments versus non-recurrent patients. Time-dependent ROC analysis demonstrates that CIN values exhibit good predictive performance between the 2nd and 5th years. High CIN was significantly associated with tumor recurrence, higher tumor grade, with high-CIN patients exhibiting poorer prognosis (median RFS: 22 months vs. unreached in the low-CIN group, p = 0.009). Multivariate analysis confirmed high CIN as an independent predictor of recurrence (HR = 5.22, 95% CI: 1.52-17.93, P = 0.009). Patients with single-chromosome copy number abnormalities also showed worse outcomes (median RFS: 16–23 months) compared to those without such abnormalities. No significant difference in RFS was detected between Ta and T1 stages (P = 0.577).

**Conclusion:**

High CIN exhibits a correlation with bladder tumor recurrence, higher tumor grade. High CIN or the presence of specific chromosomal abnormalities are indicative of an adverse prognosis. CIN may serve as a prognostic biomarker for predicting recurrence following TURBT, particularly between the 2nd and 5th years post-surgery.

## Introduction

Bladder cancer (BC) represents one of the most prevalent urological malignancies worldwide, with non-muscle-invasive bladder cancer (NMIBC) [including Ta, T1, and carcinoma *in situ* (CIS)] accounting for approximately 75% of newly diagnosed cases (1).

The primary treatment for NMIBC consists of transurethral resection of bladder tumor (TURBT), supplemented by postoperative intravesical chemotherapy or Bacillus Calmette-Guérin (BCG) immunotherapy. However, the five-year recurrence rate remains alarmingly high (31%–78%) (2). Current risk stratification relies predominantly on clinicopathological parameters including tumor stage, WHO 2004/2016 grade, tumor size, and multifocality (3), yet these exhibit limited predictive accuracy. There is an urgent need for novel biomarkers to optimize prognostic prediction.

Chromosomal instability (CIN) refers to the persistent gain or loss of large genomic segments, manifesting in two distinct forms: structural CIN and numerical CIN (4). Compared to general genetic biomarkers, CIN—characterized by chromosomal arm-level gains/losses or arm-level copy number variations—encompasses multiple oncogenes or tumor suppressor genes, offering a more macroscopic perspective on the entire genome. It reflects extensive dysregulation within tumor signaling pathways that disrupts chromosome segregation during mitosis (5). Multiple mechanisms can induce CIN, including oncogenic signaling, centrosome duplication defects, defective spindle assembly checkpoint signaling, and impaired microtubule-kinetochore attachments (4). As a hallmark feature of cancer genomes, CIN drives intratumoral heterogeneity and accelerates adaptive evolution under therapeutic pressure (6).

Recent studies demonstrate that CIN correlates with poor clinical outcomes in multiple malignancies, including colorectal (7), breast (8), pancreatic (9), and liver cancers (10). Chromosomal abnormalities involving chromosomes 3, 8, 9, 11, 13, and 17 have been identified in BC (11). Recent advances in low-coverage whole genome sequencing (LC-WGS) have enabled high-resolution, cost-effective analysis of CIN in tumor tissues (12). These technological breakthroughs provide new opportunities to dynamically monitor CIN and evaluate its clinical relevance in real time. Our earlier work identified CIN as a clinically significant prognostic biomarker in patients underwent radical cystectomy (13). However, the predictive value of CIN for post-TURBT recurrence remains insufficiently explored. Elucidating the role of CIN in predicting recurrence after TURBT may help identify candidates for intensified surveillance. Such risk stratification could enable earlier detection and timely intervention, thereby improving clinical outcomes and highlighting its clinical utility.

Studies demonstrate that LC-WGS is equally applicable to formalin-fixed paraffin-embedded (FFPE) specimens, achieving accuracy comparable to matched blood samples and yielding reliable results (14). Numerous studies employing LC-WGS for copy number analysis of DNA from FEEP specimens have been published (15, 16). This study aims to investigate the association between CIN burden derived from FFPE tissue samples and the risk of recurrence following TURBT. By integrating LC-WGS data from postoperative tumor tissues of 50 TURBT patients, we seek to establish CIN as a clinically actionable biomarker.

## Materials and methods

### Patient characteristics and ethical statement

This retrospective single-center cohort study enrolled FFPE tissue samples from 50 BC patients who underwent TURBT at the Department of Urology, Changshu Hospital Affiliated to Soochow University between December 2012 and January 2024. The inclusion criteria for this study were patients with NMIBC (Ta or T1), excluding CIS. Exclusion criteria included prior history of BC, previous intravesical therapy, non-urothelial carcinoma, incomplete follow-up, inadequate tissue/DNA samples, and low sequencing quality. Based on the EAU guidelines for NMIBC management, the post-TURBT instillation protocol is established as follows: For low-risk patients, administer a single immediate postoperative instillation of chemotherapeutic agents. For intermediate-risk patients, implement maintenance instillation with either chemotherapeutic agents or BCG for one year. For high-risk patients, employ BCG maintenance instillation for one year. The regimen for intravesical instillation of chemotherapeutic agents is once weekly for 8 doses, followed by once monthly for 10 doses. The BCG regimen comprises: Induction phase: Weekly instillation for 6 weeks. Consolidation phase: Biweekly instillation for 3 doses. Maintenance phase: Monthly instillation for 10 doses (17). All patients underwent follow-up in strict accordance with the protocol established by the EAU guidelines for NMIBC (1). The recurrence defined in this study refers to cystoscopically visible recurrence confirmed by biopsy or surgical pathology. All suspicious lesions underwent cystoscopic biopsy to determine the presence of recurrence. Follow-up data were collected until January 1, 2025. After obtaining informed consent, control samples, consisting of DNA extracted from urine sediment cells, were concurrently obtained from 11 healthy volunteers. The study protocol received approval from the Ethics Committee of Changshu Hospital Affiliated to Soochow University (Approval No. 2021-58), and all procedures complied with ethical standards. Based on Article 32 of the Declaration of Helsinki, this retrospective study utilizing FFPE tissue samples was granted an ethics waiver exempting the requirement for obtaining individual informed consent (18).

### BC pathology

Histopathological examination was performed on samples from 50 BC patients to characterize tumors. Pathological grading followed the WHO 2004/2016 classification system, with all histopathological examinations performed by a single pathologist.

### DNA extraction

Genomic DNA was isolated from FFPE tissue samples using the QIAamp DNA FFPE Tissue Kit (Qiagen; catalog no. 56404) according to the manufacturer’s instructions. The concentration of purified amplification products was quantified using a Qubit fluorometer with its corresponding kit. The calculated total library yield must meet or exceed 100.0 ng; otherwise, the library construction is invalid and requires repetition. Fragment size distribution was assessed using the Qsep series automated nucleic acid/protein analysis system. A qualified library must exhibit a primary peak between 180 bp and 1800 bp, with an average fragment size of 300.0 bp to 550.0 bp, where the primary peak constitutes ≥90.0% of the distribution. For sequencing data, each sample must yield ≥10 million reads (M = 10^6^), with ≥85% of bases achieving Q30 quality scores and ≥70% uniquely mapped reads. Failure to meet any sequencing metric necessitates re-sequencing.

### Low−coverage whole−genome sequencing

Libraries for LC-WGS were prepared from DNA inputs (50–1000 ng) using Kappa Hyper kits (Roche, California, USA) combined with custom adapters (IDT, California, USA). Library concentration and fragment size distribution were assessed via fluorescence quantification and capillary electrophoresis, respectively. Purified libraries underwent high-throughput sequencing on the Illumina HiSeq XTen platform using 150-base paired-end reads per lane. Segmental copy number alterations (CNAs) were identified using the ultrasensitive chromosomal aneuploidy detector (UCAD), a bespoke bioinformatics pipeline enabling precise detection of CNAs. The specific parameter configuration and calculation methods for UCAD were based on previously published literature (19, 20). Samples with a median absolute deviation (MAD) of the genomic log copy ratio exceeding 0.38—indicating suboptimal sequencing quality—were excluded from analysis.

### Statistical analyses

DNA was analyzed using the Illumina X10 system. Approximately 10 million paired-end reads per sample were aligned to the human reference genome (hg19) with BWA v0.7.17-r1188. Genomic coverage, assessed via the mpileup package, enabled precise read depth quantification. The average coverage for each 200-kb bin was calculated, and Z-scores normalized using the formula below.


$${\text{Z}}_{\text{bin}}={\text{coverage}}_{\text{normalized}}=\frac{{\text{coverage}}_{\text{raw}}-\text{mean}\left({\text{coverage}}_{\text{controls, raw}}\right)}{\text{stdev}\left({\text{coverage}}_{\text{controls,raw}}\right)}$$

*Z_bin_*: Standardized Z-score for a specific genomic bin; *coverage_raw_*: Raw coverage value of the bin under investigation; *coverage_controls, raw_*: Raw coverage values from control samples; *mean(coverage_controls, raw_)*: Mean raw coverage value across control samples; *stdev(coverage_controls, raw_)*: Standard deviation of raw coverage values in control samples.

Significant genomic breakpoints and copy number segments were identified using the Circular Binary Segmentation (CBS) algorithm (DNAcopy R package) (21). Categorical variables were analyzed using chi-square or Wilcoxon tests, as appropriate. The CIN score was calculated as CIN = sum(Lchr × Zchr), where Lchr is the chromosome segment length and Zchr is its Z-score. Time-dependent Receiver Operating Characteristic (ROC) analysis was performed to evaluate the predictive efficacy of CIN values for post-TURBT outcomes. The optimal cutoff value for CIN was determined using maximally selected rank statistics. Statistical significance was defined as P< 0.05.

Continuous variables conforming to a normal distribution are presented as mean ± standard deviation. Continuous variables with a skewed distribution are described using median (interquartile range). Categorical variables are expressed as frequency (percentage). Between-group comparisons for continuous variables were performed using the independent samples t-test or Mann-Whitney U test, based on their distribution characteristics. Categorical data were compared using the χ² test or Fisher’s exact test. The significance level (α) was set at 0.05 for all tests, which were two-sided. Survival analysis was conducted using Kaplan-Meier curves with log-rank testing. The proportional hazards assumption test was employed to evaluate the stability of the model. Lasso regression was used for variable selection. Cox proportional hazards regression models were used for multivariable analysis to assess the independent influence of variables on recurrence-free survival (RFS) time. The index date (“time zero”) for RFS is the date of TURBT. The concordance index (C-index) quantifies the predictive accuracy of survival models by calculating the probability that, for a randomly selected pair of subjects, the model’s predicted risk order aligns with the observed outcome order. The likelihood ratio test evaluates the goodness-of-fit between nested models by comparing the ratio of their maximized likelihood functions. Net Reclassification Improvement (NRI) assesses the incremental predictive utility of a new model versus a reference model by quantifying the net proportion of subjects reclassified correctly across risk strata.

Statistical analyses were performed using R (version 3.4.3; R Foundation for Statistical Computing), SPSS 17.0 (IBM, Foster City, CA, USA), and MedCalc (version 20.0; MedCalc Software, Mariakerke, Belgium).

## Results

### Patient characteristics

**Figure 1:**
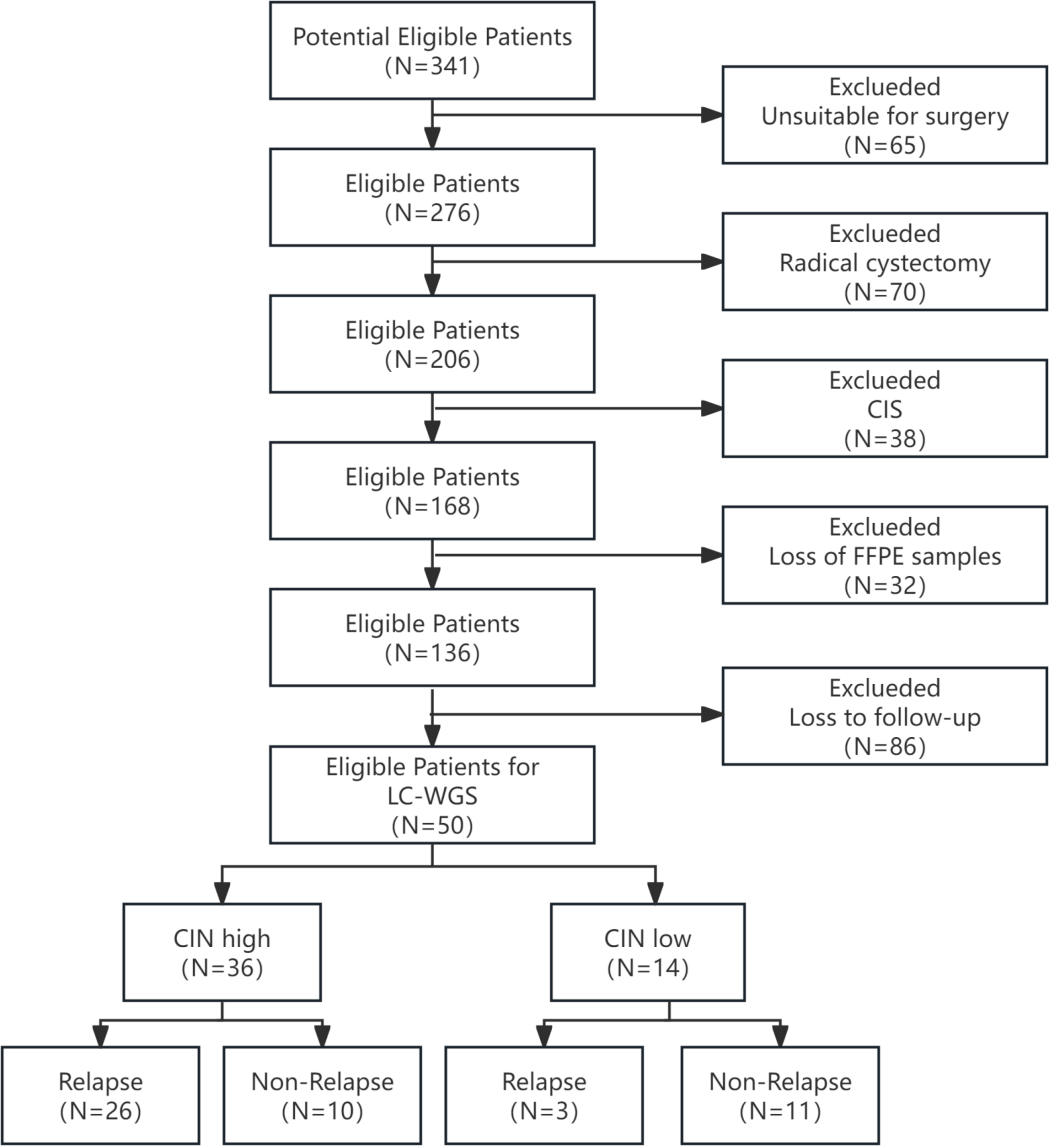
The flowchart for participant recruitment.

A total of 50 FFPE BC samples were included in this study, all of which passed quality control. **Figure 1** presents the flowchart. The mean age of the relapse group was 69.24 ± 11.43, while that of the non-relapse group was 66.33 ± 10.11, with no statistically significant difference between the two groups. Furthermore, the relapse and non-relapse groups showed no statistically significant differences in gender, smoking history, tumor size, tumor number, tumor grade, tumor stage, or composition of intravesical drugs, as detailed in **Table 1**. Most baseline covariates had standardized differences below 0.20, confirming good group comparability. Notwithstanding a standardized difference of 0.296 for intravesical drugs, this covariate was included in subsequent multivariable regression models to adjust for its potential impact on the results.

**Table 1:** Clinical features of the patients.

Patients			
Variables	Relapse	Non-relapse	P-value
	(n=29)	(n=21)	
Age	69.24±11.43	66.33±10.11	0.357
≤68 years	15(57.7%)	11(42.3%)	0.963
>68 years	14(58.3%)	10(41.7%)	
Sex			0.561
Male	23(56.10%)	18(43.90%)	
Female	6(66.67%)	3(33.33%)	
Smoking History			
No	25(64.10%)	14(35.90%)	0.100
Yes	4(36.36%)	7(63.64%)	
Tumor Size	2.99±1.24	2.51±1.14	0.175
<3 cm	13(50.0%)	13(50.0%)	0.233
≥3 cm	16(66.67%)	8(33.33%)	
Tumor count			
Single	13(52.00%)	12(48.00%)	0.390
Multiple	16(64.00%)	9(36.00%)	
Tumor Grade			0.598
Low	13(54.17%)	11(45.83%)	
High	16(61.54%)	10(38.46%)	
Tumor Stage			0.493
Ta	11(52.38%)	10(47.62%)	
T1	18(62.07%)	11(37.93%)	
Intravesical drugs			0.255
BCG	4(50.00%)	4(50.00%)	
Gemcitabine	18(58.06%)	13(41.94%)	
Hydroxycamptothecin	3(100%)	0(0%)	
Mitomycin	2(33.33%)	4(66.67%)	
Pirarubicin	2(100%)	0(0%)	

BCG, Bacillus Calmette–Guérin.

### Cancer genome of BC

**Figure 2:**
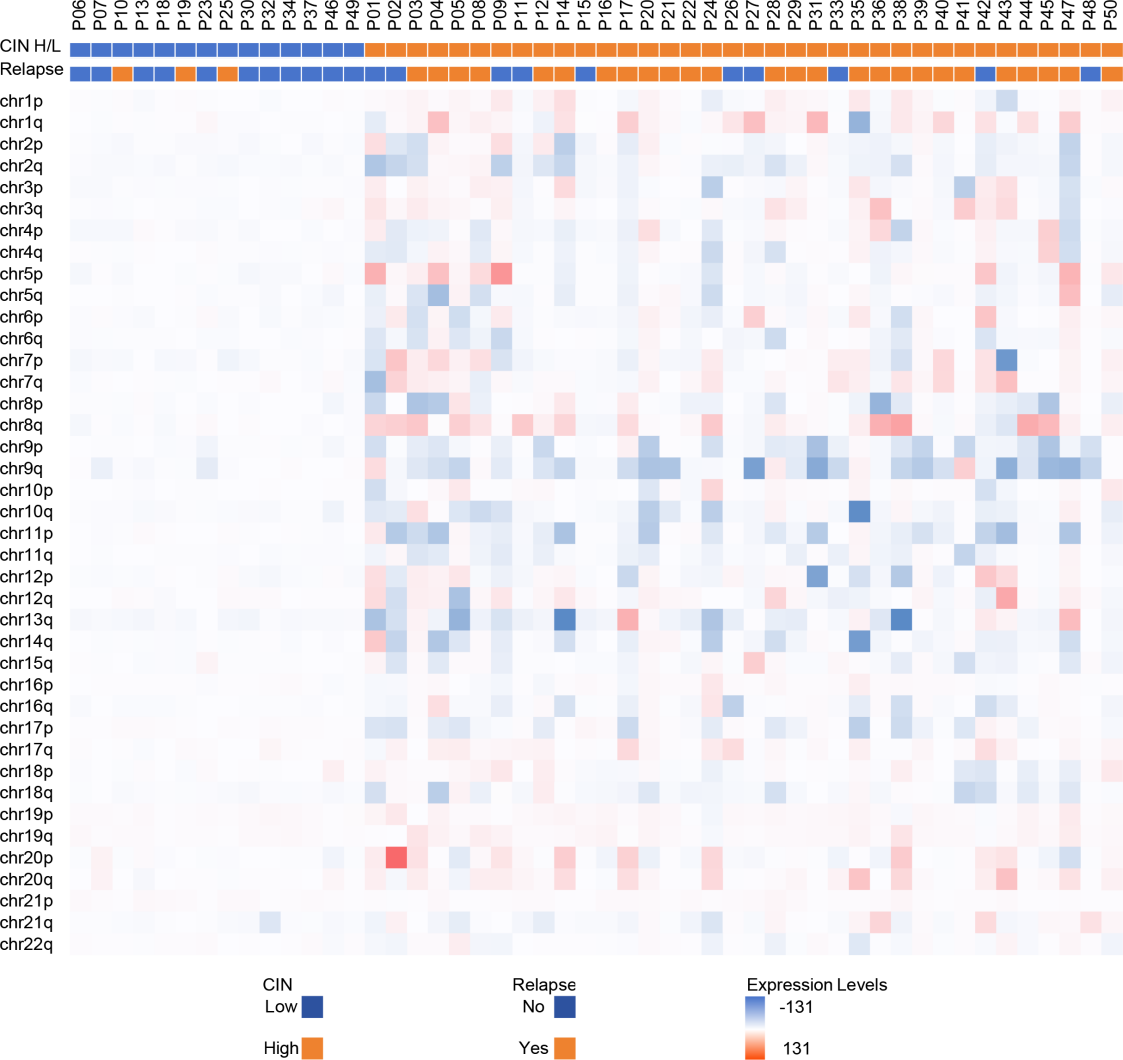
Heatmap of chromosomal instability (CIN) profiles across bladder cancer patients stratified by relapse and CIN status. The color of each row indicates changes in chromosomal segments (red, copy gains; blue, copy losses).

The median coverage for all samples was 0.497 (interquartile range: Q1 = 0.422, Q3 = 0.558)(**Supplementary Table 1**). Comparative analysis revealed statistically significant CNAs in 67 gene segments of BC patients with recurrence relative to non-recurrence cases (**Table 2**). Hierarchical clustering of sample-level CNAs demonstrated distinct genomic patterns in the heatmap (**Figure 2**). Relapse group samples (orange) exhibited intensified red-blue signals, indicating marked CIN, whereas non-relapse counterparts (blue) showed attenuated variations, consistent with enhanced genomic stability.

**Table 2:** Copy number alterations of genome segments identified in bladder cancer.

Chromosome	Loc.Start	Loc.End	Seg.Mean	log10(P)^a^	Key Genes
chr9	71,000,000	141,000,000	-4.58	-175.2	
chr11	0	47,800,000	-3.70	-141.7	
chr14	61,800,000	107,000,000	-2.95	-131.3	
chr9	0	39,000,000	-3.61	-103.6	*CDKN2A*
chr17	0	21,600,000	-3.45	-62.6	*TP53*
chr11	103,400,000	134,800,000	-2.52	-60.9	
chr8	8,200,000	33,200,000	-2.90	-58.2	*DLC1*
chr10	0	39,000,000	0.38	-57.4	
chr8	66,600,000	99,400,000	2.03	-52.1	*YWHAZ*
chr20	24,800,000	55,000,000	2.20	-50.7	
chr7	44,800,000	158,800,000	0.62	-41.2	*EGFR*
chr15	20,000,000	102,200,000	-0.93	-40.6	
chr5	132,600,000	180,600,000	-1.39	-33.0	
chr4	172,400,000	189,800,000	-2.14	-30.4	
chr10	97,000,000	135,200,000	-2.32	-29.5	*FGFR2*
chr8	103,800,000	109,200,000	2.69	-29.3	
chr3	44,000,000	89,600,000	-0.77	-28.5	*FHIT*
chr16	10,200,000	48,600,000	0.74	-26.9	
chr14	19,000,000	61,600,000	-1.46	-26.9	
chr12	0	28,200,000	-1.15	-25.1	
chr18	21,200,000	71,000,000	-1.76	-25.0	
chr6	200,000	88,000,000	-0.29	-24.4	
chr3	89,800,000	197,800,000	0.81	-24.3	*PIK3CA*
chr8	38,800,000	66,400,000	1.30	-23.0	
chr10	83,800,000	96,200,000	-2.28	-22.3	*PTEN*
chr19	0	58,800,000	0.93	-21.9	*KEAP1*
chr5	0	40,200,000	0.03	-19.7	*TERT*
chr5	50,000,000	132,400,000	-0.77	-19.0	
chr16	48,800,000	90,000,000	-0.86	-16.9	
chr1	118,800,000	187,800,000	1.85	-16.3	*NTRK1*
chr1	200,000	29,600,000	0.12	-15.2	
chr20	55,200,000	62,800,000	1.17	-14.0	*BCL2L1*
chr13	41,600,000	114,800,000	-1.80	-13.7	*RB1*
chr8	140,800,000	146,000,000	3.18	-12.1	
chr8	99,600,000	103,600,000	5.03	-11.9	
chr1	188,000,000	249,000,000	0.75	-11.9	
chr8	109,400,000	118,000,000	0.94	-11.3	
chr4	108,000,000	172,200,000	-0.77	-9.3	
chr11	68,200,000	70,400,000	4.38	-8.9	*CCND1*
chr8	118,200,000	132,800,000	2.53	-8.8	*MYC*
chr11	48,000,000	68,000,000	0.43	-7.8	
chr10	42,800,000	83,600,000	-1.09	-7.7	
chr8	200,000	6,800,000	-3.12	-7.7	
chr12	71,000,000	114,800,000	0.36	-7.2	
chr21	19,400,000	28,400,000	-1.43	-7.1	
chr2	0	207,600,000	-0.78	-6.2	*NFE2L2*
chr22	36,000,000	51,000,000	-1.70	-6.0	
chr18	71,200,000	76,200,000	-3.54	-6.0	
chr1	30,800,000	53,400,000	0.80	-4.9	
chr10	42,200,000	42,600,000	6.76	-4.2	
chr4	48,200,000	55,000,000	1.82	-4.1	
chr1	53,600,000	118,600,000	0.06	-3.7	
chr12	69,000,000	70,800,000	7.14	-3.6	*MDM2*
chr8	133,000,000	140,600,000	1.05	-3.6	
chr4	0	48,000,000	-0.52	-3.5	*FGFR3*
chr8	33,400,000	38,600,000	-0.77	-3.4	
chr4	190,000,000	190,800,000	0.18	-2.8	
chr17	21,800,000	80,800,000	0.71	-2.8	*ERBB2*
chr13	19,000,000	41,400,000	-0.32	-2.4	
chr7	0	44,600,000	-0.41	-2.1	
chr9	40,000,000	70,800,000	-0.11	-1.8	
chr12	115,000,000	120,400,000	2.09	-1.7	
chr22	16,000,000	35,800,000	-0.18	-1.7	
chr21	9,400,000	19,200,000	0.06	-1.5	
chr16	0	10,000,000	-0.32	-1.5	
chr11	70,600,000	103,200,000	-0.70	-1.4	
chr12	28,400,000	68,800,000	-0.05	-1.3	

*CDKN2A*, Cyclin dependent kinase inhibitor 2 A; *TP53*, Tumor Protein p53; *DLC1*, Deleted in liver cancer-1; *YWHAZ*, Tyrosine 3-monooxygenase/tryptophan 5-monooxygenase activation protein zeta; *EGFR*, Epidermal growth factor receptor; FGFR2, Fibroblast growth factor receptor 2; *FHIT*, The fragile histidine triad; *PIK3CA*, Phosphatidylinositol-4,5-bisphosphate 3-kinase; *PTEN*, Phosphatase and tensin homolog deleted on chromosome 10; *KEAP1*, kelch-like ECH-associated protein 1; *TERT*, Telomerase reverse transcriptase; *NTRK1*, Neurotrophic tyrosine kinase receptor 1; BCL2L1,B-cell lymphoma 2 like 1; *RB1*,Retinoblastoma 1; *CCND1*,Cyclin D1; *MYC*,v-myc avian myelocytomatosis viral oncogene homolog; *NFE2L2*, Nuclear Factor, Erythroid 2 Like 2; *MDM2*, Mouse Double Minute 2; *FGFR3*, Fibroblast Growth Factor Receptor 3; *ERBB2*, Erb-B2 Receptor Tyrosine Kinase 2.

a log10(P) < −1.3 corresponds to P < 0.05.

**Figure 3:**
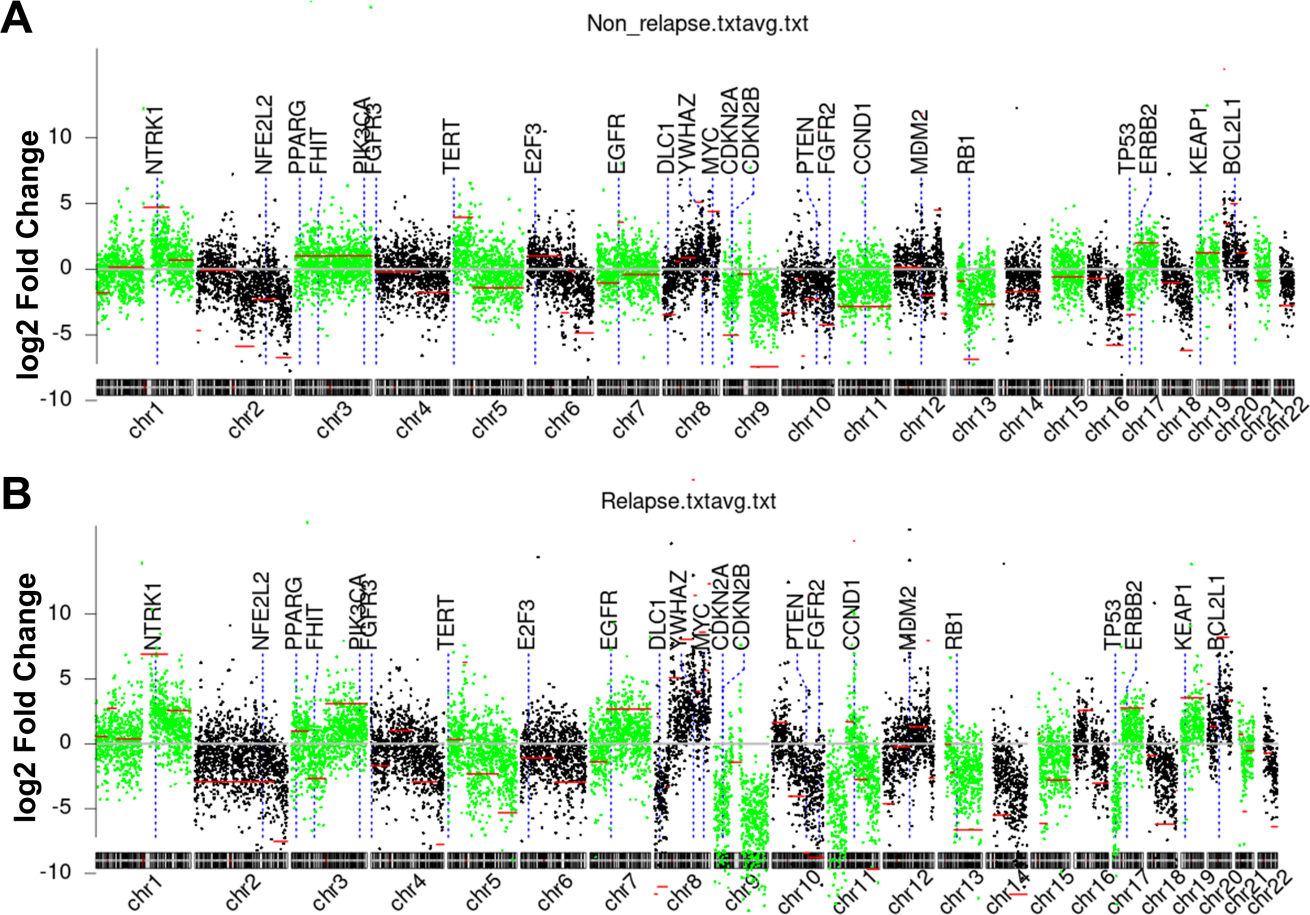
Chromosomal instability (CIN) in bladder tumor tissue. **(A)** Non-relapsed patients; **(B)** Relapsed patients.

**Supplementary Table 2** details CIN scores and chromosome-specific abnormalities across tumors. In this study, a CIN cutoff value of 6486.722 was established, with samples above this threshold classified as high CIN and those below classified as low CIN. Among the 50 BC samples, 36 (72.0%) demonstrated high CIN and 14 (28.0%) exhibited low CIN (**Table 3**). Genome-wide CNA landscapes are summarized in **Figure 3**. Relapse patients (**Figure 3B**) displayed significantly broader CIN dispersion across chromosomal regions, indicating pervasive genomic instability. Notably, relapse cohorts harbored prominent CIN outliers at loci of key oncogenes [e.g., Tumor Protein p53 (*TP53*), Cyclin dependent kinase inhibitor 2 A (*CDKN2A*), Epidermal growth factor receptor (*EGFR*)], suggesting their mechanistic involvement in recurrence. Amplification (green) and deletion (negative value) events also occurred more frequently in this group genome-wide. Conversely, non-relapse cases (**Figure 3A**) manifested constrained CIN distributions with minimal fluctuations, reflecting structural stability.

**Table 3:** Clinicopathologial characteristics and CIN of 50 bladder cancer patients samples.

CIN				
Variables	High	Low	Total	P-value
	(n=36)	(n=14)		
CIN	23672.44(13114.66,38664.64)	3200.80(1737.12,3829.94)	14973.90(4242.72,31184.90)	**<0.001**
Age	68.39±11.73	67.07±8.66	68.02±10.89	0.705
≤68 years	20(76.92%)	6(23.08%)	26	0.420
>68 years	16(66.67%)	8(33.33%)	24	
Gender				1.000*
Female	7(77.78%)	2(22.22%)	9	
Male	29(70.73%)	12(29.27%)	41	
Smoking History				0.476*
No	29(74.36%)	10(25.64%)	39	
Yes	7(63.64%)	4(36.36%)	11	
Tumor size (cm)				0.086
<3	16(61.54%)	10(38.46%)	26	
≥3	20(83.33%)	4(16.67%)	24	
Tumor count				1.000
Single	18(72.00%)	7(28.00%)	25	
Multiple	18(72.00%)	7(28.00%)	25	
Tumor grade				**0.039**
Low	14(58.33%)	10(41.67%)	24	
High	22(84.62%)	4(15.38%)	26	
Tumor Stage				0.939
Ta	15(71.43%)	6(28.57%)	21	
T1	21(72.41%)	8(27.59%)	29	
Relapse				**0.001**
Yes	26(89.66%)	3(10.34%)	29	
No	10(47.62%)	11(52.38%)	21	

CIN, chromosomal instability; BCG, Bacillus Calmette–Guérin.

*Fisher’s exact test.

Values in bold indicate statistical significance.

### Association of molecular subtypes with tumor stage and survival outcome

**Figure 4:**
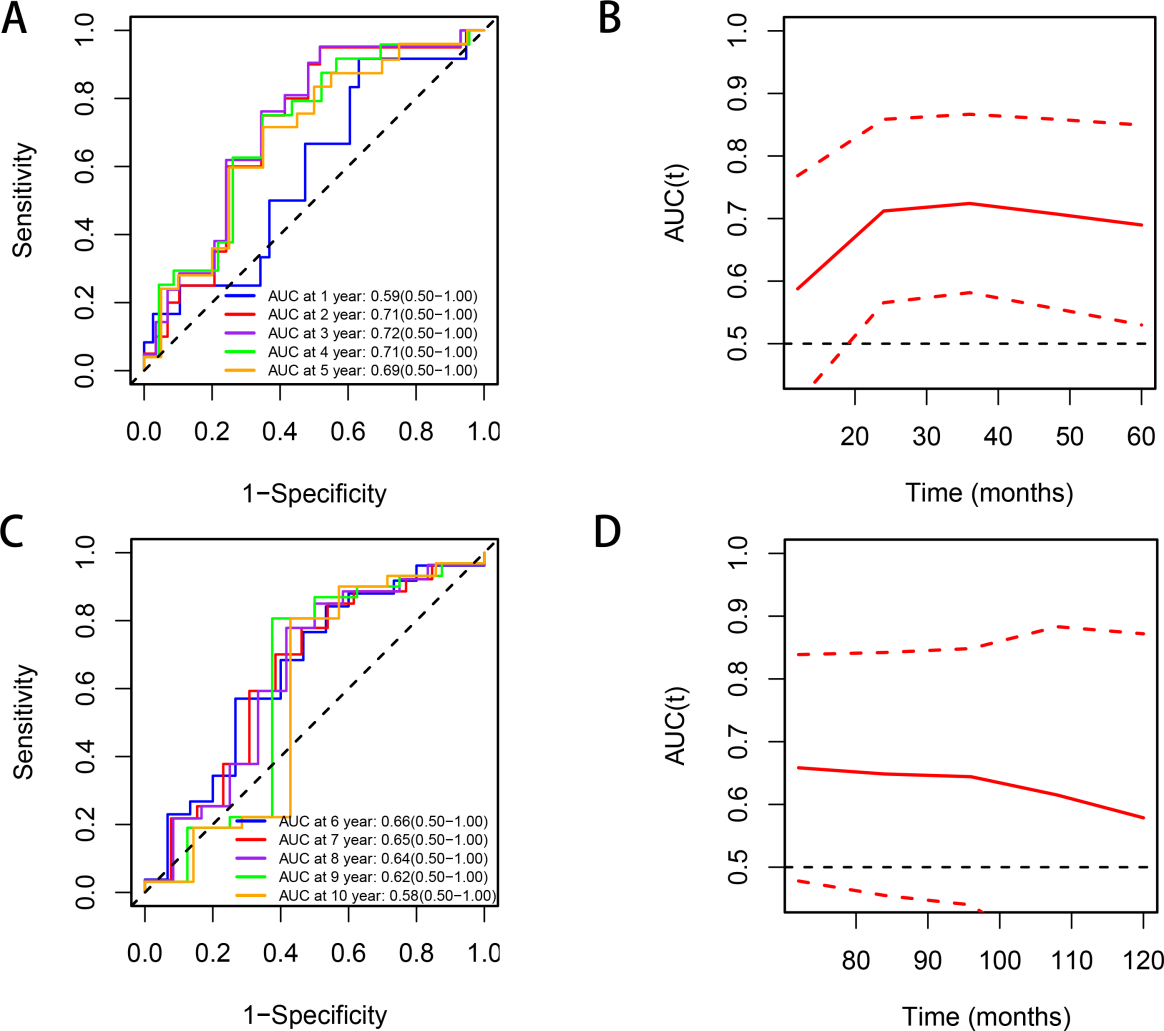
Time-dependent ROC curves and AUC values (with 95% confidence bands) of the CIN score for predicting relapse. **(A, B)** years 1–5; **(C, D)** years 6–10.

Time-dependent receiver operating characteristic (ROC) analysis demonstrates that CIN values exhibit good predictive performance between the 2nd and 5th years, with AUC values around 0.7. Beyond the 6th year, the AUC gradually declines to approximately 0.6 (**Figure 4**).

As detailed in **Table 3**, molecular classification of tumor tissue was based on CIN levels. High CIN was associated with higher tumor grade and increased recurrence rate (P = 0.039 and P = 0.001, respectively), but showed no significant associations with age, gender, smoking history, tumor size, tumor stage or tumor count (all P > 0.05).

### Association of CIN and tumor stage with RFS

**Figure 5:**
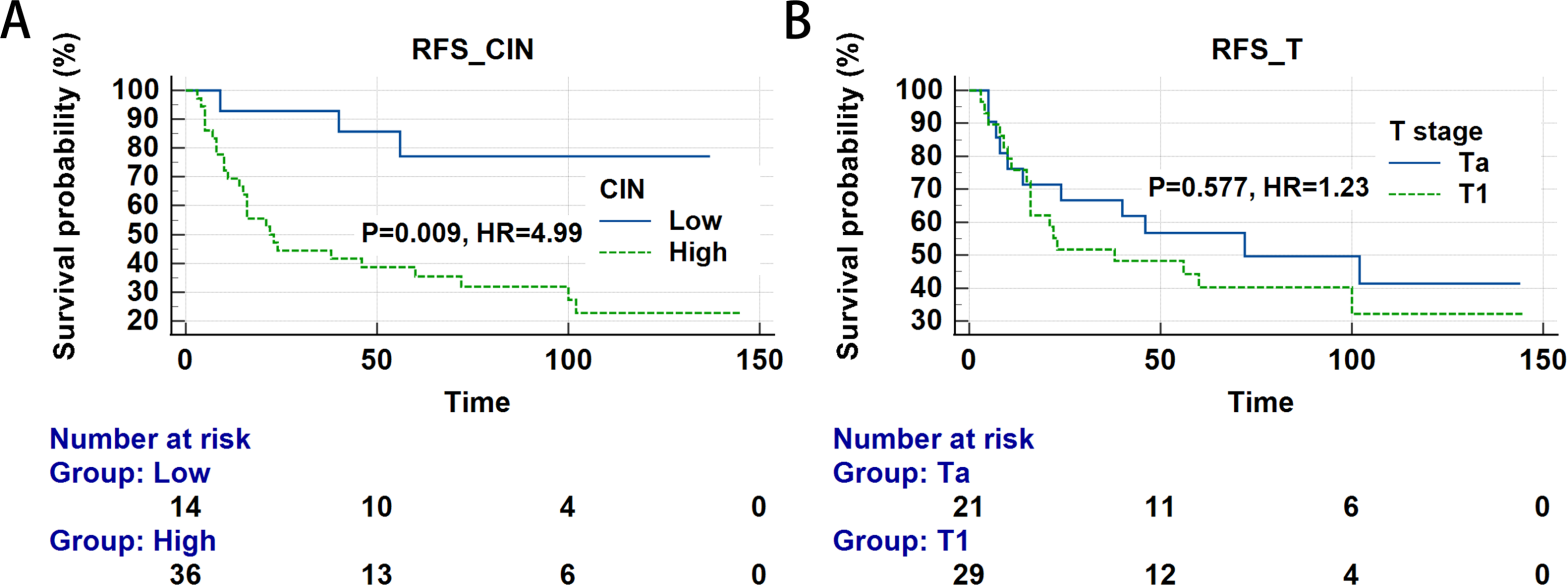
Relationship between chromosomal instability (CIN), T stage and survival of patients with bladder cancer (Time measured in months). **(A)** recurrence-free survival (RFS) for CIN; **(B)** RFS for T staging.

Our analysis examined RFS across molecular subtypes. **Figure 5A** illustrates significantly distinct survival outcomes among these subtypes (Logrank test, P = 0.009). Specifically, the high CIN group exhibited a reduced RFS rate, with a median survival of 22 months, while low CIN patients demonstrated improved RFS. Patients with high CIN faced a significantly elevated relapse risk compared to the low CIN group (HR = 4.99, 95% CI: 1.51-16.54, P = 0.009; **Table 4**; **Figure 5A**). No significant difference in RFS was detected between Ta and T1 stages (HR = 1.23, 95% CI: 0.59-2.57, P = 0.577; **Table 4**; **Figure 5B**).

**Table 4:** The relationship between CIN and Tumor stage and RFS in bladder cancer patients.

Variables	Group	Relapse		Median		P-value	HR (95%CI)
		Yes	No	RFS			
CIN	low	3	11	–	–		Reference
	high	26	10	22	**0.009**		**4.99 (1.51-16.54)**
Tumor stage	Ta	11	10	72			Reference
	T1	18	11	38	0.577		1.23 (0.59-2.57)

CIN, chromosomal instability; RFS, recurrence-free survival.

Values in bold indicate statistical significance.

Utilizing Lasso regression analysis, we identified CIN as the sole covariate. Consequently, we excluded covariates such as age, gender, tumor size, and tumor number. Multivariate Cox regression analysis, incorporating tumor grade, intravesical drugs, and CIN level (high/low) as covariates, revealed that high CIN was an independent prognostic factor for RFS (HR = 5.22, 95% CI: 1.52-17.93, P = 0.009)(**Table 5**). We used the proportional hazards assumption test to evaluate the stability of the model. The results showed that the p-values for the covariates tumor grade, intravesical drugs, and CIN level (high/low) were 0.433, 0.635, and 0.908, respectively, and the global test p-value was 0.797, indicating that the model is stable and reliable.

**Table 5:** Univariate and multivariate analyses of factors associated with relapse of patients.

Variables	Univariate		Multivariate	
	HR (95%CI)	P-value	HR (95%CI)	P-value
Tumor grading				
Low	Reference		Reference	
High	1.23 (0.59-2.57)	0.577	0.89 (0.42-1.91)	0.766
Intravesical drugs				
BCG	Reference		Reference	
non-BCG drugs	1.19 (0.83-1.70)	0.336	1.19 (0.82-1.73)	0.360
CIN				
Low	Reference		Reference	
High	**4.99 (1.51-16.54)**	**0.009**	**5.22 (1.52-17.93)**	**0.009**

CIN, chromosomal instability; BCG, Bacillus Calmette–Guéri.

Values in bold indicate statistical significance.

We further compared the predictive performance of the EAU risk stratification for NMIBC and CIN for recurrence. The prognostic model based on the new European Association of Urology prognostic-factor risk groups for non–muscle-invasive BC using either the WHO 2004/2016 or WHO 1973 grading classification system achieved a C-index of 0.573. In contrast, the prognostic prediction model constructed using the CIN classification yielded a superior C-index of 0.635. The likelihood ratio test demonstrated that incorporating the CIN grouping variable into the EAU prognostic risk model significantly improved model fit (chi-square value=9.503, df=1, P = 0.002). With a NRI of 0.704, the CIN grouping variable substantially enhanced the predictive capability of the model.

### Prognostic value of the Z chromosome score for survival outcomes

**Figure 6:**
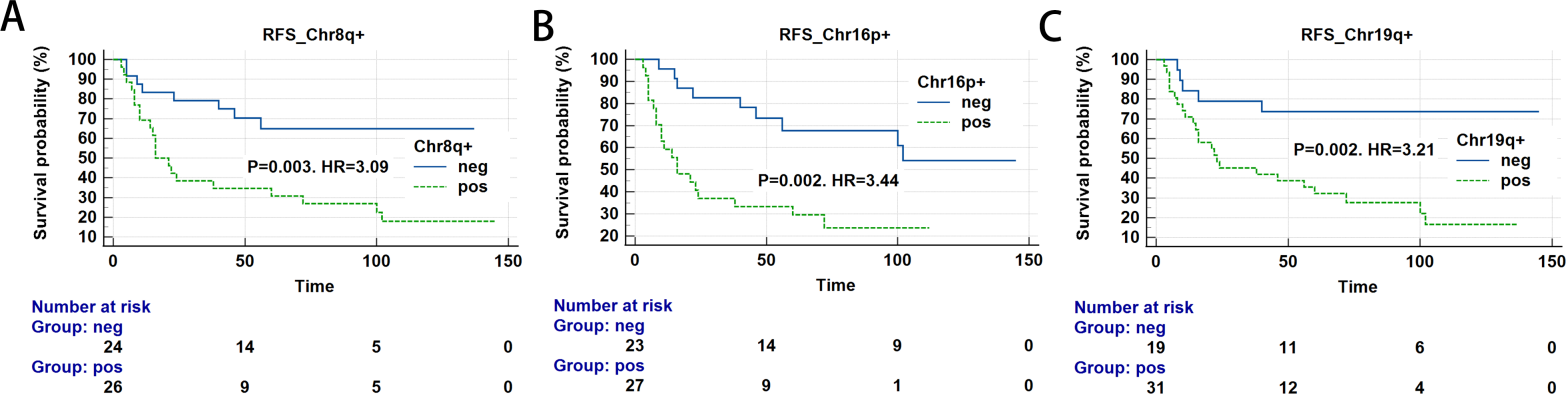
Single chromosomal arm copy number variations (CNVs) associated with worse recurrence-free survival (RFS)(Time measured in months). **(A)** RFS for chr8q+; **(B)** RFS for chr16p+; **(C)** RFS for chr19q+.

To assess the prognostic value of Z chromosome score in BC survival, we calculated their predictive performance. Chromosome arms 16p, 19q, and 8q demonstrated the highest predictive accuracy, with AUCs of 0.691, 0.691, and 0.666, respectively (**Table 6**). We subsequently evaluated RFS in patients exhibiting amplification or deletion of single chromosome arms. Patients harboring specific CNAs—gains in 8q+, 16p+, 19q+ experienced significantly poorer outcomes, evidenced by a median RFS of 16 to 23 months. In contrast, patients without these specific alterations exhibited markedly longer RFS (**Table 7**; **Figure 6**).

**Table 6:** Predictive efficacy of chromosome Z scores for relapse in bladder cancer FFPE samples.

Chromosome	AUC	P-value	Z score cutoff	95% CI for AUC
chr16p	0.691	**0.006**	1.568	**[0.525-0.835]**
chr19q	0.691	**0.016**	3.227	**[0.520-0.828]**
chr8q	0.666	**0.007**	2.371	**[0.494-0.813]**

CHR, chromosome; AUC, area under curve; FFPE, formalin-fixed paraffin-embedded.

Values in bold indicate statistical significance.

**Table 7:** Relationships between abnormal copy number of a single chromosome and RFS of bladder cancer patients.

Chromosome	Status	Relapse		Median RFS	P-value	HR (95%CI)
		Yes	No			
8q+	negative	8	16	–	–	Reference
	positive	21	5	16	**0.003**	**3.09 (1.47-6.49)**
16p+	negative	9	14	–		Reference
	positive	20	7	16	**0.002**	**3.44 (1.60-7.38)**
19q+	negative	5	14	–	–	Reference
	positive	24	7	23	**0.002**	**3.21 (1.53-6.73)**

RFS, recurrence-free survival .

Values in bold indicate statistical significance.

## Discussion

The study found that high CIN was significantly associated with tumor recurrence and higher tumor grade, correlating with poorer RFS. Multivariate analysis identified high CIN as an independent predictor of recurrence. Patients exhibiting single-chromosome copy number abnormalities also showed worse outcomes compared to those without such abnormalities. No significant difference in RFS was observed between Ta stage and T1 stage. These findings suggest that CIN may serve as a prognostic biomarker for predicting post-TURBT recurrence. Based on our research findings, we propose conducting LC-WGS on tumor tissues obtained through TURBT to determine the degree of CIN. Using the calculated cut-off value, patients can be categorized into high-CIN or low-CIN groups. As patients with high CIN face significantly higher recurrence risks, they require close monitoring and more aggressive treatment strategies.

Time-dependent ROC analysis demonstrated that the CIN score achieved optimal predictive performance for relapse between years 2 and 5 (AUC ≈ 0.7), but declined thereafter to approximately 0.6 beyond year 6. This decline may be attributed to tumor clonal evolution over time, where late recurrences are driven by acquired mutations rather than baseline CIN; a potential “cured” population in whom baseline features no longer dictate outcome; and statistical attrition due to censoring. Clinically, CIN is most useful as a medium-term prognostic indicator (years 2–5) to guide early surveillance intensity, but is unsuitable for ultra-long-term prediction, where risk assessment should be updated with contemporary findings. External validation in larger cohorts is warranted to confirm these temporal patterns.

CIN reflects the dysregulation of multiple tumor-related pathways affecting chromosome distribution during mitosis (5). The manifestations of CIN are diverse, including lagging chromosomes, chromosome bridges, micronuclei, aneuploidy, and polyploidy (22–24). The root causes of CIN are errors during DNA replication, resulting in the formation of cells with incomplete or excessive genetic material; or errors in chromosome segregation during cell division, leading to the formation of cells with an aberrant chromosome number (24). The presence of chromosomal aberrations in over 90% of human tumors suggests widespread CIN in cancer (25). Multiple studies have demonstrated an association between CIN and higher tumor grade, with CIN being significantly elevated in patients experiencing tumor recurrence and metastasis (26–28). Consistent with these reports, the findings of this study also show that patients with high CIN have higher tumor grade and significantly increased tumor recurrence.

A significantly higher prevalence of CIN was observed in recurrent BC cases. The selection of treatment regimens for patients adheres to the EAU risk stratification groups. In our region, due to the high cost of BCG and lack of insurance coverage, some patients diagnosed with high-grade urothelial carcinoma opted for chemotherapeutic instillation therapy instead due to economic constraints. Subsequently, regression analysis incorporated tumor grade, intravesical drugs, and CIN level (high/low) as covariates and revealed that high CIN significantly increased the risk of tumor recurrence.

In this study, we identified specific CNAs in BC, encompassing chromosomal aberrations such as1q+, 7p+, 8p-, 9p-, 9q-, 17p-, and 17q+. These aberrations involve genomic regions harboring genes with established roles in BC pathogenesis, including *CDKN2A* (9p), *TP53* (17p), and *EGFR* (7p). Deletion of chromosome 9, frequently observed in low-grade and early-stage BC, has been linked to higher recurrence rates, particularly involving loss at 9q22.3, 9q33, and 9q34 (29, 30). Loss of 8p, often affecting the secreted frizzled-related protein 1 (*SFRP1*) gene at 8p12–11.1, is associated with invasive tumor behavior, higher stage and grade, and shorter overall survival (31). Conversely, gain of 1q, including upregulation of proline rich coiled-coil 2C (*PRRC2C*) in the 1q23–24 region, correlates with increased invasiveness (32). Polysomy of chromosomes 7 and 17 is associated with high p53 expression, which may promote tumor proliferation and progression (33), and has been shown to confer a 3.62-fold higher risk of recurrence in NMIBC (34). Notably, copy number loss of *CDKN2A*—one of the most frequent CNAs in BC—correlates with reduced gene expression (35) and has been associated with adverse prognosis in NMIBC (36). Likewise, *EGFR* amplification or overexpression participates in signaling pathways linked to tumor progression and metastasis (37, 38). *TP53* missense mutations, for instance, have been implicated as early drivers in bladder carcinogenesis (39). Collectively, these findings suggest that the genomic instability captured by our CIN metric reflects underlying disruptions in key oncogenic pathways, thereby providing a biological basis for its notable association with tumor recurrence following TURBT.

While the prognostic model based on the EAU risk stratification for NMIBC demonstrated limited predictive ability (C-index: 0.573), the CIN-based classification showed superior performance (C-index: 0.635). Likelihood ratio test confirmed that incorporating CIN significantly improved model fit (χ²=9.503, df=1, P = 0.002), with a substantial net reclassification improvement (NRI = 0.704), underscoring its incremental prognostic value. Although the predictive efficacy of CIN alone was modest (C-index = 0.635), it remained an independent risk factor in multivariable analysis and contributed significantly to NRI of the combined model, indicating that it provides incremental information in the integrated model. CIN possesses potential prognostic value and warrants further validation in larger cohorts.

Multiple studies have demonstrated a correlation between CIN and prognosis in cancer patients. In a study of 354 patients with hormone receptor-positive and human epidermal growth factor receptor 2-negative breast cancer, those with high CIN exhibited shorter invasive disease-free survival and overall survival (OS). High CIN was identified as an independent prognostic predictor (8). Li et al. investigated 91 patients with initially resectable colorectal cancer liver metastases who underwent curative liver resection and found that high CIN was associated with poorer 3-year RFS and lower 3-year OS (40). In another study, Chen et al. analyzed FFPE tumor tissue samples from 91 pancreatic cancer patients and demonstrated that high CIN, along with the presence of human herpesvirus (*HHV*)*-7* and *HHV-5* DNA, was correlated with worse prognosis (9). Consistent with the aforementioned studies, our findings revealed that patients with high CIN exhibited higher recurrence rates. Notably, these patients demonstrated significantly shorter RFS. Multivariate regression analysis identified high CIN as an independent risk factor for predicting RFS. No statistically significant differences in recurrence rates or RFS were observed between Ta and T1 stage patients, though this finding may reflect limitations due to the relatively small cohort size, potentially introducing selection bias. However, in a study of 40 gastric cancer patients, Ye et al. reported that patients with high CIN had a poorer prognosis, while no statistically significant survival difference was observed across different traditional Borrmann types (21). Their findings, together with our results, suggest that CIN may be, to some extent, superior to conventional pathological classification in prognostic assessment. This warrants further validation through multicenter studies with larger sample sizes.

Current studies have consistently demonstrated that individual chromosomal abnormalities are associated with unfavorable prognosis in various cancers. Alshalalfa et al. reported that in prostate cancer patients, CNAs in Chr8q and overexpression of related genes correlate with higher Gleason scores, increased metastatic risk, and elevated prostate cancer-specific mortality (41). Multi-omics analyses of Pan-Cancer datasets from The Cancer Genome Atlas (TCGA) and the Genomic Data Commons (GDC) revealed that segmental copy number loss and somatic mutations at chromosome 1p36.13 are linked to poorer prognosis in primary tumors (42). In stage III colorectal cancer, loss of chromosome 4q leads to downregulated expression of phosphoribosylaminoimidazole succinocarboxamide synthetase and is associated with worse RFS (43). Similarly, our study confirms the correlation between individual chromosomal abnormalities and tumor recurrence, with chr16p+, chr19q+, and chr8q+ demonstrating certain prognostic value. This study revealed that chr16p+, chr19q+, and chr8q+ are associated with worse RFS, as detailed in **Tables 6** and **7**. Tyrosine 3-monooxygenase/tryptophan 5-monooxygenase activation protein zeta (*YWHAZ*), located at 8q22.3, has been shown to be closely associated with BC development (44) and correlates with higher tumor stage, lymphovascular invasion, and mitotic activity (45). Kelch-like ECH-associated protein 1 (*KEAP1*) is situated on chromosome 19q. The KEAP1 and NRF2 (nuclear factor-E2-related factor 2) signaling pathway can be activated by p62, protecting BC cells from oxidative stress damage and thereby promoting tumor growth (46). GWAS studies indicate that a polymorphism in the cyclin E1 protein (*CCNE1*) gene locus at 19q12 correlates with elevated cyclin E protein expression, which accelerates the cell cycle and enhances BC invasiveness (47). However, no cancer-related genes located specifically on 16p have yet been identified in the context of BC pathogenesis. Further mechanistic studies are required to elucidate potential drivers in this region. The identification of key driver chromosomal abnormalities in tumor pathogenesis enables more precise assessment of recurrence risk at reduced costs.

This study has the following strengths. First, it is the first to investigate the predictive value of CIN and individual chromosomal abnormalities for recurrence in BC patients following TURBT. Second, no significant differences were observed in baseline characteristics (age, sex, tumor size, tumor number, tumor stage) between the recurrence and non-recurrence groups, ensuring comparability. Several limitations should be noted. First, the relatively small sample size and single-center design limit the statistical power and may introduce bias. Second, the specific mechanisms underlying the influence of individual chromosomal abnormalities on BC prognosis were not elucidated. Future studies are needed to investigate these underlying biological mechanisms. Third, the cutoff value is data-driven and requires validation through subsequent multi-center studies with larger sample sizes to confirm our findings. Fourth, it should be acknowledged that there was considerable heterogeneity between the two groups regarding the intravesical agents administered. Although logistic regression was employed to adjust for this potential confounder, the non-randomized nature of the study means that residual confounding from treatment selection bias may persist. Lastly, the study characterized by its prolonged enrollment period, inherently carries the biases and confounding issues typical of retrospective research. Furthermore, temporal changes in clinical practice have compounded data heterogeneity and complicated causal inference, potentially compromising the robustness and temporal validity of the conclusions.

## Conclusions

CIN was significantly associated with tumor recurrence and higher tumor grade of BC. Patients with high CIN or specific single-chromosome copy number abnormalities demonstrated poorer RFS. High CIN was identified as an independent predictor of recurrence and may serve as a prognostic biomarker for predicting recurrence following TURBT, particularly between the 2nd and 5th years post-surgery.

## Data Availability

The original contributions presented in the study are included in the article/**Supplementary Material**. Further inquiries can be directed to the corresponding author.
